# (*E*)-4-Meth­oxy-2-[3-(trifluoro­meth­yl)phenyl­imino­meth­yl]phenol

**DOI:** 10.1107/S160053680905034X

**Published:** 2009-11-28

**Authors:** Zeynep Keleşoğlu, Orhan Büyükgüngör, Çiğdem Albayrak, Mustafa Odabaşoğlu

**Affiliations:** a Department of Physics, Ondokuz Mayıs University, TR-55139 Samsun, Turkey; b Sinop University, Sinop Faculty of Education, Sinop, Turkey; c Chemistry Program, Denizli Higher Vocational School, Pamukkale University, TR-20159 Denizli, Turkey

## Abstract

The title compound, C_15_H_12_F_3_NO_2_, adopts the phenol–imine tautomeric form, with the H atom attached to oxygen rather than to nitro­gen. There are two independent mol­ecules aligned nearly parallel in the asymmetric unit with their trifloramethyl groups pointing in opposite directions. The dihedral angles between the aromatic rings are 40.43 (1)° in the first mol­ecule and 36.12 (1)° in the second. Strong intra­molecular O—H⋯N hydrogen bonding generates *S*(6) ring motifs. Weak inter­molecular C—H⋯O hydrogen bonds link the independent mol­ecules separately into sheets normal to [010]. In addition, C—H⋯π inter­actions are also observed. The F atoms of the trifluoro­methyl groups are disordered over two sets of sites with refined site occupancies of 0.59 (2)/0.41 (2) and 0.62 (3)/0.38 (3), respectively.

## Related literature

For the photochromic and thermochromic characteristics of Schiff base compounds, see: Williams (1972 ▶); Calligaris *et al.* (1972 ▶); Gavronic *et al.* (1996 ▶); Hadjoudis *et al.* (1987 ▶). For graph-set motifs, see: Bernstein *et al.* (1995 ▶). For related structures, see: Temel *et al.* (2007 ▶); Odabaşoğlu & Büyükgüngör (2006 ▶).
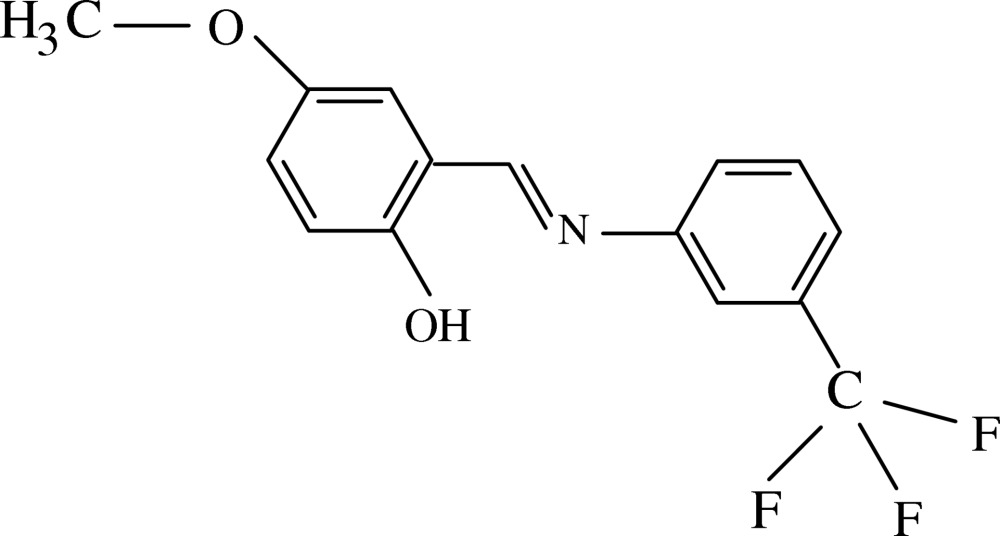


## Experimental

### 

#### Crystal data

C_15_H_12_F_3_NO_2_
*M* *_r_* = 295.26
Monoclinic, 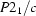

*a* = 13.4771 (7) Å
*b* = 6.4526 (2) Å
*c* = 31.7097 (15) Å
β = 92.647 (4)°
*V* = 2754.6 (2) Å^3^
*Z* = 8
Mo *K*α radiation
μ = 0.12 mm^−1^
*T* = 296 K
0.80 × 0.43 × 0.15 mm

#### Data collection

Stoe IPDS II diffractometer
Absorption correction: integration (*X-RED32*; Stoe & Cie, 2002 ▶) *T*_min_ = 0.739, *T*_max_ = 0.944
23526 measured reflections
5197 independent reflections
3536 reflections with *I* > 2σ(*I*)
*R*_int_ = 0.075

#### Refinement

*R*[*F* ^2^ > 2σ(*F* ^2^)] = 0.065
*wR*(*F* ^2^) = 0.205
*S* = 1.07
5197 reflections
444 parameters
144 restraints
H atoms treated by a mixture of independent and constrained refinement
Δρ_max_ = 0.20 e Å^−3^
Δρ_min_ = −0.25 e Å^−3^

### 

Data collection: *X-AREA* (Stoe & Cie, 2002 ▶); cell refinement: *X-AREA*; data reduction: *X-RED32* (Stoe & Cie, 2002 ▶); program(s) used to solve structure: *SHELXS97* (Sheldrick, 2008 ▶); program(s) used to refine structure: *SHELXL97* (Sheldrick, 2008 ▶); molecular graphics: *ORTEP-3 for Windows* (Farrugia, 1997 ▶); software used to prepare material for publication: *WinGX* (Farrugia, 1999 ▶) and *PLATON* (Spek, 2009 ▶).

## Supplementary Material

Crystal structure: contains datablocks I, global. DOI: 10.1107/S160053680905034X/si2222sup1.cif

Structure factors: contains datablocks I. DOI: 10.1107/S160053680905034X/si2222Isup2.hkl

Additional supplementary materials:  crystallographic information; 3D view; checkCIF report

## Figures and Tables

**Table 1:** Hydrogen-bond geometry (Å, °)

*D*—H⋯*A*	*D*—H	H⋯*A*	*D*⋯*A*	*D*—H⋯*A*
O1—H1⋯N1	0.91 (4)	1.79 (4)	2.619 (4)	150 (4)
O1*A*—H1*A*⋯N1*A*	0.87 (4)	1.87 (4)	2.623 (3)	143 (4)
C10—H10⋯O1^i^	0.93	2.58	3.444 (3)	154
C10*A*—H10*A*⋯O1*A* ^i^	0.93	2.54	3.413 (3)	157
C3—H3⋯*Cg*3^ii^	0.93	2.86	3.526 (3)	130
C3*A*—H3*A*⋯*Cg*1^ii^	0.93	2.88	3.518 (3)	127
C11—H11⋯*Cg*4^iii^	0.93	2.85	3.529 (3)	131
C11*A*—H11*A*⋯*Cg*2^iii^	0.93	2.97	3.646 (3)	131

Symmetry codes: (i) 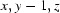; (ii) 
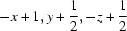
; (iii) 
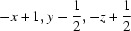
. *Cg*1, *Cg*2, *Cg*3 and *Cg*4 are the centroids of the C1–C6, C9-C14, C1*A*–C6*A* and C9*A*–C14*A* rings, respectively.

**Fig. 1.**
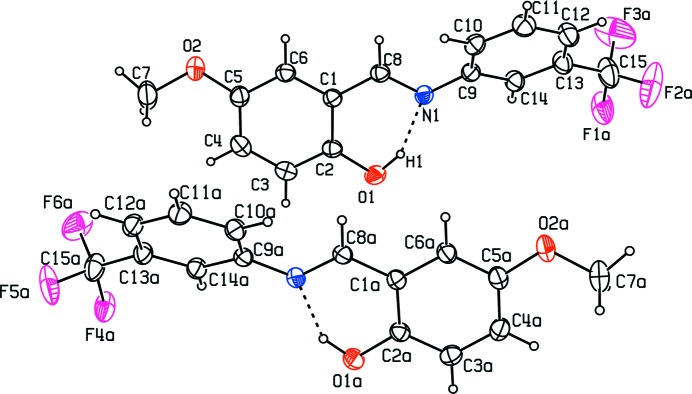
An ORTEP view of (I), with the atom-numbering scheme and 20% probability displacement ellipsoids. The minor disorder components of the trifluoromethyl F atoms were omitted. Dashed lines indicate H-bonds.

**Fig. 2.**
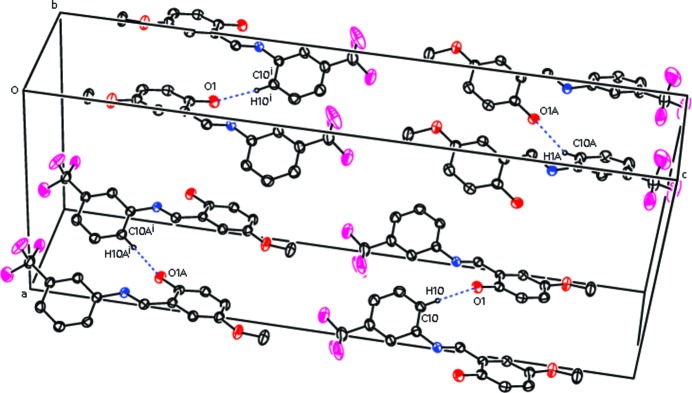
A packing diagram for (I), showing the C—H···O hydrogen bonds. H atoms not involved in hydrogen bonding (dashed lines) have been omitted for clarity. [Symmetry codes; (i): x,-1 + y,z].

**Fig. 3.**
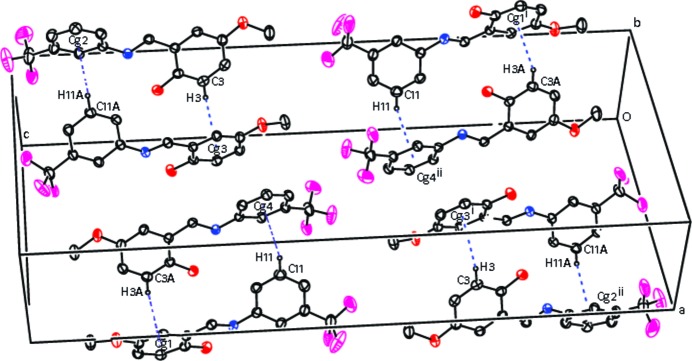
A packing diagram for (I), showing the C—H···π interactions. H atoms not involved in hydrogen bonding (dashed lines) have been omitted for clarity. [Symmetry codes; (i): 1 - x, 1/2 + y, 1/2 - z; (ii): 1 - x, -1/2 + y, 1/2 - z]. (Cg1,Cg2 and Cg3, Cg4 are the centroids of the C1—C6, C9—C14; C1A—C6A, C9A—C14A rings, respectively).

## supplementary crystallographic information

### Comment

Most Schiff bases have antibacterial, anticancer, antinflammatory and antitoxic properties (Williams, 1972). In addition to that, Schiff bases have been used widely as ligands in the field of coordination chemistry (Calligaris *et al.*, 1972). The Schiff base compounds can be classified by their photochromic and thermochromic characteristics (Hadjoudis *et al.*, 1987).

Photochromism is produced by an intramolecular proton transfer associated with a change in the π-electron configuration. Studies on photochromic compounds have been increasing ever since the potential applications of photochromic materials were realised in various areas, such as the control and measurement of radiation intensity, optical computers and display systems. Two types of intramolecular hydrogen bonds [either N—H···O (keto form) or N···H—O (enol form)] can exist in Schiff bases. The Schiff bases derived from salicyaldehyde always form the N···H–O type of hydrogen bonding, regardless of the nature of the N substituent (alkyl or aryl) (Gavronic *et al.*, 1996).

The asymmetric unit of (I) contains two independent molecules aligned in opposite direction (Fig. 1.) and intermolecular hydrogen bonds C10—H10···O1 and C10A—H10A···O1A linked both independent molecules separately into sheets along [010] (Table 1. and Fig. 2.). The similar packing were observed in the structure (*E*)-3-[2-(Trifluoromethyl)phenyliminomethyl]-benzene-1,2-diol (Temel *et al.*, 2007) but with O—H···O intermolecular hydrogen bonds. Intramolecular O—H···N hydrogen bonds generating S(6) ring motif (Bernstein *et al.*, 1995) are observed in both molecules. The two mutual aromatic rings of the molecules in the asymmetric unit inclined at 2.56 (2)° and 12.37 (12)°. The dihedral angles between the two benzene rings are 40.43 (1)° in the first molecule and 36.12 (1)° in the second molecule numbered with label A.

The crystal packing is also stabilized by C11—H11···*Cg*4, C3A—H3A···*Cg*1 and C11A—H11A···*Cg*2 π-ring interactions (Fig.3, Table 1). Similar results were observed in 3-[3-(Trifluoromethyl)anilino]isobenzofuran-1(3*H*)-one (Odabaşoğlu & Büyükgüngör (2006).

The CF3 group shows rotational disorder; the F atoms of the trifluoromethyl groups are disordered over two positions with refined site occupancies of 0.59 (2)/0.41 (2) and 0.62 (3)/0.38 (3), respectively.

### Experimental

The compound(I) was prepared by stirring for 1 h under reflux, the mixture of 5-methoxysalicylaldehyde (0.5 g, 3.3 mmol) in ethanol (20 ml) and 3-trifluoromethylaniline (0.53 g, 3.3 mmol) in ethanol (20 ml). The crystals suitable for X-ray analysis were obtained from methanol by slow evaporation (yield; 74%, m.p.; 344–345 K).

### Refinement

The hydroxyl H atoms were located in difference Fourier map and were refined freely. All other H-atoms were refined using a riding model with d(C—H) = 0.93Å (*U*_iso_=1.2*U*_eq_ of the parent atom) for aromatic C atoms and d(C—H) = 0.96Å (*U*_iso_=1.5*U*_eq_ of the parent atom) for methyl C atoms. The CF3 group shows rotational disorder with occupancy factors of 0.59 (2)/0.41 (2) and 0.62 (3)/0.38 (3) for both molecules in the asymmetric unit. Similar *U_ij_* and isotropic *U_ij_* restraints applied to these F atoms. The bond distances of C—F were fixed to 1.346 Å with 0.02 e.s.d. in the refinement.

### Crystal data

**Table:**

C_15_H_12_F_3_NO_2_	*F*(000) = 1216
*M**_r_* = 295.26	*D*_x_ = 1.424 Mg m^−^^3^
Monoclinic, *P*2_1_/*c*	Mo *K*α radiation, λ = 0.71073 Å
Hall symbol: -P 2ybc	Cell parameters from 20067 reflections
*a* = 13.4771 (7) Å	θ = 1.3–25.7°
*b* = 6.4526 (2) Å	µ = 0.12 mm^−^^1^
*c* = 31.7097 (15) Å	*T* = 296 K
β = 92.647 (4)°	Prism, yellow
*V* = 2754.6 (2) Å^3^	0.80 × 0.43 × 0.15 mm
*Z* = 8	

### Data collection

**Table:**

Stoe IPDS II diffractometer	5197 independent reflections
Radiation source: fine-focus sealed tube	3536 reflections with *I* > 2σ(*I*)
plane graphite	*R*_int_ = 0.075
Detector resolution: 6.67 pixels mm^-1^	θ_max_ = 25.7°, θ_min_ = 1.3°
rotation method scans	*h* = −16→16
Absorption correction: integration (*X-RED32*; Stoe & Cie, 2002)	*k* = −7→7
*T*_min_ = 0.739, *T*_max_ = 0.944	*l* = −38→38
23526 measured reflections	

### Refinement

**Table:**

Refinement on *F*^2^	Secondary atom site location: difference Fourier map
Least-squares matrix: full	Hydrogen site location: inferred from neighbouring sites
*R*[*F*^2^ > 2σ(*F*^2^)] = 0.065	H atoms treated by a mixture of independent and constrained refinement
*wR*(*F*^2^) = 0.205	*w* = 1/[σ^2^(*F*_o_^2^) + (0.0875*P*)^2^ + 1.1131*P*] where *P* = (*F*_o_^2^ + 2*F*_c_^2^)/3
*S* = 1.07	(Δ/σ)_max_ < 0.001
5197 reflections	Δρ_max_ = 0.20 e Å^−^^3^
444 parameters	Δρ_min_ = −0.25 e Å^−^^3^
144 restraints	Extinction correction: *SHELXL97* (Sheldrick, 2008), Fc^*^=kFc[1+0.001xFc^2^λ^3^/sin(2θ)]^-1/4^
Primary atom site location: structure-invariant direct methods	Extinction coefficient: 0.0018 (5)

### Special details

**Table:**

Geometry. All e.s.d.'s (except the e.s.d. in the dihedral angle between two l.s. planes) are estimated using the full covariance matrix. The cell e.s.d.'s are taken into account individually in the estimation of e.s.d.'s in distances, angles and torsion angles; correlations between e.s.d.'s in cell parameters are only used when they are defined by crystal symmetry. An approximate (isotropic) treatment of cell e.s.d.'s is used for estimating e.s.d.'s involving l.s. planes.
Refinement. Refinement of *F*^2^ against ALL reflections. The weighted *R*-factor *wR* and goodness of fit *S* are based on *F*^2^, conventional *R*-factors *R* are based on *F*, with *F* set to zero for negative *F*^2^. The threshold expression of *F*^2^ > σ(*F*^2^) is used only for calculating *R*-factors(gt) *etc*. and is not relevant to the choice of reflections for refinement. *R*-factors based on *F*^2^ are statistically about twice as large as those based on *F*, and *R*- factors based on ALL data will be even larger.

### Fractional atomic coordinates and isotropic or equivalent isotropic displacement parameters (Å^2^)

**Table:**

	*x*	*y*	*z*	*U*_iso_*/*U*_eq_	Occ. (<1)
F1A	0.1216 (9)	0.4505 (8)	0.46713 (17)	0.126 (3)	0.592 (15)
F2A	0.1877 (10)	0.1908 (17)	0.5018 (2)	0.155 (4)	0.592 (15)
F3A	0.0307 (7)	0.218 (2)	0.4785 (4)	0.187 (5)	0.592 (15)
F1B	0.1963 (14)	0.406 (2)	0.4751 (4)	0.165 (5)	0.408 (15)
F2B	0.1215 (13)	0.1269 (16)	0.5008 (3)	0.126 (4)	0.408 (15)
F3B	0.0359 (10)	0.324 (3)	0.4722 (6)	0.181 (6)	0.408 (15)
C1A	0.61622 (19)	0.2839 (4)	0.27472 (8)	0.0492 (6)	
C2A	0.6470 (2)	0.4936 (5)	0.27532 (9)	0.0522 (7)	
C3A	0.6768 (2)	0.5824 (5)	0.31314 (9)	0.0596 (7)	
H3A	0.6973	0.7201	0.3137	0.072*	
C4A	0.6770 (2)	0.4718 (5)	0.35037 (10)	0.0629 (8)	
H4A	0.6974	0.5352	0.3756	0.075*	
C5A	0.6471 (2)	0.2668 (5)	0.35018 (9)	0.0607 (8)	
C6A	0.6160 (2)	0.1754 (5)	0.31277 (8)	0.0564 (7)	
H6A	0.5944	0.0385	0.3128	0.068*	
C7A	0.6930 (4)	0.2192 (8)	0.42341 (11)	0.1045 (14)	
H7A1	0.6875	0.1179	0.4454	0.157*	
H7A2	0.6605	0.3448	0.4314	0.157*	
H7A3	0.7618	0.2469	0.4192	0.157*	
C8A	0.59249 (19)	0.1754 (5)	0.23556 (8)	0.0517 (7)	
H8A	0.5768	0.0352	0.2365	0.062*	
C9A	0.5837 (2)	0.1543 (4)	0.16155 (8)	0.0502 (6)	
C10A	0.6170 (2)	−0.0492 (5)	0.15797 (9)	0.0561 (7)	
H10A	0.6420	−0.1193	0.1818	0.067*	
C11A	0.6127 (2)	−0.1466 (5)	0.11934 (10)	0.0631 (8)	
H11A	0.6342	−0.2831	0.1174	0.076*	
C12A	0.5771 (2)	−0.0451 (5)	0.08346 (10)	0.0636 (8)	
H12A	0.5743	−0.1118	0.0574	0.076*	
C13A	0.5455 (2)	0.1581 (5)	0.08704 (9)	0.0596 (7)	
C14A	0.5483 (2)	0.2566 (5)	0.12565 (9)	0.0567 (7)	
H14A	0.5263	0.3927	0.1276	0.068*	
C15A	0.5104 (3)	0.2720 (5)	0.04844 (10)	0.0829 (11)	
N1A	0.59247 (17)	0.2674 (4)	0.19956 (7)	0.0529 (6)	
O1A	0.64877 (18)	0.6071 (4)	0.23935 (7)	0.0691 (6)	
O2A	0.6472 (2)	0.1424 (4)	0.38544 (7)	0.0873 (8)	
C1	0.10504 (19)	0.2748 (5)	0.24086 (9)	0.0527 (7)	
C2	0.1374 (2)	0.4839 (5)	0.23806 (9)	0.0564 (7)	
C3	0.1381 (2)	0.5767 (5)	0.19883 (11)	0.0649 (8)	
H3	0.1589	0.7136	0.1968	0.078*	
C4	0.1089 (2)	0.4713 (6)	0.16290 (11)	0.0692 (9)	
H4	0.1098	0.5373	0.1369	0.083*	
C5	0.0776 (2)	0.2655 (6)	0.16503 (10)	0.0642 (8)	
C6	0.0749 (2)	0.1719 (5)	0.20390 (9)	0.0584 (7)	
H6	0.0523	0.0361	0.2055	0.070*	
C7	0.0736 (3)	0.2186 (9)	0.09038 (11)	0.1054 (15)	
H7A	0.0501	0.1211	0.0694	0.158*	
H7B	0.1443	0.2333	0.0892	0.158*	
H7C	0.0424	0.3505	0.0852	0.158*	
C8	0.1080 (2)	0.1653 (5)	0.28078 (9)	0.0549 (7)	
H8	0.0905	0.0258	0.2812	0.066*	
C9	0.1463 (2)	0.1420 (5)	0.35335 (9)	0.0559 (7)	
C10	0.1841 (2)	−0.0589 (5)	0.35468 (10)	0.0620 (8)	
H10	0.1978	−0.1267	0.3297	0.074*	
C11	0.2011 (3)	−0.1575 (6)	0.39286 (11)	0.0741 (9)	
H11	0.2256	−0.2922	0.3935	0.089*	
C12	0.1821 (3)	−0.0587 (6)	0.42978 (11)	0.0795 (10)	
H12	0.1941	−0.1258	0.4555	0.095*	
C13	0.1451 (3)	0.1413 (6)	0.42881 (10)	0.0749 (9)	
C14	0.1274 (2)	0.2419 (5)	0.39071 (9)	0.0655 (8)	
H14	0.1028	0.3766	0.3902	0.079*	
C15	0.1269 (5)	0.2510 (8)	0.46891 (13)	0.1131 (16)	
N1	0.13402 (17)	0.2560 (4)	0.31550 (7)	0.0555 (6)	
O1	0.16842 (17)	0.5928 (4)	0.27249 (8)	0.0710 (6)	
O2	0.0501 (2)	0.1466 (5)	0.13076 (7)	0.0893 (8)	
F4A	0.5051 (12)	0.4750 (10)	0.0524 (3)	0.089 (2)	0.62 (3)
F5A	0.5747 (12)	0.2314 (18)	0.0164 (3)	0.109 (3)	0.62 (3)
F6A	0.4221 (8)	0.220 (2)	0.0334 (4)	0.126 (3)	0.62 (3)
F4B	0.5467 (18)	0.466 (2)	0.0490 (6)	0.094 (4)	0.38 (3)
F5B	0.5269 (19)	0.191 (2)	0.0124 (3)	0.104 (4)	0.38 (3)
F6B	0.4096 (8)	0.275 (4)	0.0467 (8)	0.137 (6)	0.38 (3)
H1	0.162 (3)	0.506 (7)	0.2948 (13)	0.095 (14)*	
H1A	0.627 (3)	0.535 (7)	0.2175 (13)	0.091 (13)*	

### Atomic displacement parameters (Å^2^)

**Table:**

	*U*^11^	*U*^22^	*U*^33^	*U*^12^	*U*^13^	*U*^23^
F1A	0.211 (8)	0.094 (4)	0.074 (3)	0.016 (4)	0.022 (4)	−0.028 (2)
F2A	0.204 (8)	0.175 (7)	0.083 (4)	0.046 (6)	−0.034 (4)	−0.028 (4)
F3A	0.230 (8)	0.181 (8)	0.159 (7)	−0.003 (6)	0.113 (6)	−0.048 (6)
F1B	0.216 (10)	0.161 (9)	0.120 (6)	−0.031 (8)	0.029 (7)	−0.066 (6)
F2B	0.189 (9)	0.140 (7)	0.051 (4)	0.014 (6)	0.029 (5)	0.005 (4)
F3B	0.219 (10)	0.174 (11)	0.154 (8)	0.067 (8)	0.059 (7)	−0.026 (8)
C1A	0.0495 (14)	0.0506 (16)	0.0478 (14)	0.0021 (12)	0.0037 (11)	0.0036 (12)
C2A	0.0495 (14)	0.0529 (17)	0.0542 (16)	0.0006 (12)	0.0020 (11)	0.0071 (13)
C3A	0.0552 (16)	0.0545 (18)	0.0692 (19)	−0.0009 (13)	0.0029 (13)	−0.0075 (14)
C4A	0.0592 (17)	0.076 (2)	0.0533 (16)	−0.0008 (15)	−0.0001 (13)	−0.0100 (15)
C5A	0.0610 (17)	0.072 (2)	0.0489 (15)	0.0024 (15)	0.0060 (12)	0.0053 (14)
C6A	0.0654 (17)	0.0543 (17)	0.0500 (15)	−0.0006 (14)	0.0063 (12)	0.0068 (13)
C7A	0.131 (4)	0.130 (4)	0.052 (2)	0.001 (3)	−0.009 (2)	0.008 (2)
C8A	0.0520 (15)	0.0523 (17)	0.0507 (15)	−0.0048 (12)	0.0012 (11)	0.0065 (12)
C9A	0.0506 (14)	0.0516 (16)	0.0480 (14)	−0.0060 (12)	−0.0010 (11)	0.0061 (12)
C10A	0.0616 (17)	0.0522 (17)	0.0539 (16)	0.0013 (13)	−0.0029 (12)	0.0081 (13)
C11A	0.076 (2)	0.0478 (17)	0.0650 (18)	0.0028 (15)	−0.0039 (14)	0.0017 (14)
C12A	0.081 (2)	0.0553 (19)	0.0534 (16)	−0.0049 (16)	−0.0049 (14)	−0.0045 (14)
C13A	0.079 (2)	0.0516 (18)	0.0479 (15)	0.0000 (15)	−0.0057 (13)	0.0042 (13)
C14A	0.0707 (18)	0.0469 (17)	0.0521 (15)	0.0007 (14)	−0.0017 (13)	0.0047 (12)
C15A	0.132 (4)	0.060 (2)	0.0557 (19)	0.008 (2)	−0.015 (2)	−0.0002 (16)
N1A	0.0560 (13)	0.0553 (14)	0.0470 (12)	−0.0017 (11)	−0.0007 (10)	0.0053 (10)
O1A	0.0904 (16)	0.0550 (14)	0.0612 (13)	−0.0100 (11)	−0.0048 (11)	0.0140 (11)
O2A	0.117 (2)	0.098 (2)	0.0461 (12)	−0.0097 (15)	−0.0007 (12)	0.0132 (12)
C1	0.0472 (14)	0.0555 (18)	0.0560 (16)	−0.0006 (12)	0.0074 (11)	−0.0035 (13)
C2	0.0504 (15)	0.0551 (18)	0.0643 (18)	−0.0013 (13)	0.0082 (12)	−0.0015 (14)
C3	0.0597 (17)	0.0587 (19)	0.077 (2)	−0.0005 (14)	0.0098 (15)	0.0070 (16)
C4	0.0597 (18)	0.080 (2)	0.0683 (19)	0.0061 (16)	0.0077 (14)	0.0163 (18)
C5	0.0538 (16)	0.080 (2)	0.0587 (17)	−0.0008 (16)	0.0008 (13)	−0.0015 (16)
C6	0.0547 (16)	0.0586 (18)	0.0622 (17)	−0.0046 (13)	0.0052 (13)	0.0003 (14)
C7	0.110 (3)	0.150 (4)	0.057 (2)	−0.004 (3)	0.002 (2)	0.000 (2)
C8	0.0522 (15)	0.0533 (17)	0.0596 (17)	−0.0017 (13)	0.0062 (12)	−0.0023 (13)
C9	0.0528 (15)	0.0599 (19)	0.0553 (16)	−0.0041 (14)	0.0065 (12)	−0.0040 (14)
C10	0.0619 (17)	0.0594 (19)	0.0648 (18)	0.0021 (14)	0.0035 (14)	−0.0109 (15)
C11	0.079 (2)	0.063 (2)	0.081 (2)	0.0049 (17)	0.0050 (17)	0.0017 (18)
C12	0.096 (3)	0.075 (2)	0.067 (2)	0.004 (2)	0.0064 (18)	0.0115 (18)
C13	0.093 (2)	0.076 (2)	0.0571 (18)	0.0038 (19)	0.0165 (16)	−0.0019 (16)
C14	0.078 (2)	0.0609 (19)	0.0581 (17)	0.0026 (16)	0.0119 (14)	−0.0062 (14)
C15	0.169 (5)	0.110 (4)	0.062 (2)	0.017 (4)	0.020 (3)	0.005 (2)
N1	0.0572 (13)	0.0584 (15)	0.0511 (13)	−0.0012 (11)	0.0063 (10)	−0.0062 (11)
O1	0.0809 (15)	0.0589 (14)	0.0735 (15)	−0.0107 (11)	0.0076 (12)	−0.0116 (12)
O2	0.1061 (19)	0.109 (2)	0.0523 (13)	−0.0176 (16)	−0.0021 (12)	−0.0022 (13)
F4A	0.143 (6)	0.060 (3)	0.063 (3)	0.019 (3)	−0.010 (4)	0.0089 (19)
F5A	0.159 (7)	0.117 (5)	0.052 (3)	0.019 (4)	0.010 (3)	0.020 (3)
F6A	0.149 (6)	0.120 (6)	0.102 (5)	−0.017 (4)	−0.072 (4)	0.019 (4)
F4B	0.137 (9)	0.065 (5)	0.077 (5)	−0.011 (5)	−0.020 (7)	0.023 (4)
F5B	0.158 (10)	0.091 (6)	0.059 (4)	0.013 (6)	−0.028 (5)	−0.012 (4)
F6B	0.151 (8)	0.134 (10)	0.118 (10)	0.021 (6)	−0.064 (6)	0.019 (7)

### Geometric parameters (Å, °)

**Table:**

F1A—C15	1.290 (6)	C15A—F6A	1.306 (7)
F2A—C15	1.352 (7)	C15A—F4A	1.318 (7)
F3A—C15	1.362 (9)	C15A—F4B	1.345 (9)
F1B—C15	1.376 (9)	C15A—F6B	1.358 (10)
F2B—C15	1.295 (7)	C15A—F5A	1.389 (7)
F3B—C15	1.323 (10)	O1A—H1A	0.87 (4)
C1A—C6A	1.395 (4)	C1—C6	1.391 (4)
C1A—C2A	1.415 (4)	C1—C2	1.422 (4)
C1A—C8A	1.448 (4)	C1—C8	1.449 (4)
C2A—O1A	1.356 (3)	C2—O1	1.348 (4)
C2A—C3A	1.372 (4)	C2—C3	1.381 (4)
C3A—C4A	1.379 (4)	C3—C4	1.369 (5)
C3A—H3A	0.9300	C3—H3	0.9300
C4A—C5A	1.383 (5)	C4—C5	1.396 (5)
C4A—H4A	0.9300	C4—H4	0.9300
C5A—C6A	1.373 (4)	C5—O2	1.367 (4)
C5A—O2A	1.377 (4)	C5—C6	1.375 (4)
C6A—H6A	0.9300	C6—H6	0.9300
C7A—O2A	1.417 (4)	C7—O2	1.412 (4)
C7A—H7A1	0.9600	C7—H7A	0.9600
C7A—H7A2	0.9600	C7—H7B	0.9600
C7A—H7A3	0.9600	C7—H7C	0.9600
C8A—N1A	1.287 (3)	C8—N1	1.282 (4)
C8A—H8A	0.9300	C8—H8	0.9300
C9A—C14A	1.382 (4)	C9—C14	1.383 (4)
C9A—C10A	1.393 (4)	C9—C10	1.393 (4)
C9A—N1A	1.410 (3)	C9—N1	1.411 (4)
C10A—C11A	1.376 (4)	C10—C11	1.377 (5)
C10A—H10A	0.9300	C10—H10	0.9300
C11A—C12A	1.380 (4)	C11—C12	1.367 (5)
C11A—H11A	0.9300	C11—H11	0.9300
C12A—C13A	1.385 (4)	C12—C13	1.383 (5)
C12A—H12A	0.9300	C12—H12	0.9300
C13A—C14A	1.378 (4)	C13—C14	1.383 (5)
C13A—C15A	1.486 (4)	C13—C15	1.486 (6)
C14A—H14A	0.9300	C14—H14	0.9300
C15A—F5B	1.285 (9)	O1—H1	0.91 (4)
			
C6A—C1A—C2A	118.7 (3)	C6—C1—C8	119.9 (3)
C6A—C1A—C8A	119.3 (3)	C2—C1—C8	121.4 (3)
C2A—C1A—C8A	121.8 (2)	O1—C2—C3	119.2 (3)
O1A—C2A—C3A	119.6 (3)	O1—C2—C1	121.9 (3)
O1A—C2A—C1A	121.4 (3)	C3—C2—C1	118.8 (3)
C3A—C2A—C1A	119.0 (3)	C4—C3—C2	121.4 (3)
C2A—C3A—C4A	121.4 (3)	C4—C3—H3	119.3
C2A—C3A—H3A	119.3	C2—C3—H3	119.3
C4A—C3A—H3A	119.3	C3—C4—C5	120.5 (3)
C3A—C4A—C5A	120.1 (3)	C3—C4—H4	119.7
C3A—C4A—H4A	119.9	C5—C4—H4	119.7
C5A—C4A—H4A	119.9	O2—C5—C6	116.7 (3)
C6A—C5A—O2A	116.1 (3)	O2—C5—C4	124.5 (3)
C6A—C5A—C4A	119.5 (3)	C6—C5—C4	118.8 (3)
O2A—C5A—C4A	124.4 (3)	C5—C6—C1	121.8 (3)
C5A—C6A—C1A	121.3 (3)	C5—C6—H6	119.1
C5A—C6A—H6A	119.4	C1—C6—H6	119.1
C1A—C6A—H6A	119.4	O2—C7—H7A	109.5
O2A—C7A—H7A1	109.5	O2—C7—H7B	109.5
O2A—C7A—H7A2	109.5	H7A—C7—H7B	109.5
H7A1—C7A—H7A2	109.5	O2—C7—H7C	109.5
O2A—C7A—H7A3	109.5	H7A—C7—H7C	109.5
H7A1—C7A—H7A3	109.5	H7B—C7—H7C	109.5
H7A2—C7A—H7A3	109.5	N1—C8—C1	121.5 (3)
N1A—C8A—C1A	121.9 (3)	N1—C8—H8	119.2
N1A—C8A—H8A	119.1	C1—C8—H8	119.2
C1A—C8A—H8A	119.1	C14—C9—C10	119.3 (3)
C14A—C9A—C10A	118.9 (3)	C14—C9—N1	117.8 (3)
C14A—C9A—N1A	118.0 (3)	C10—C9—N1	122.7 (3)
C10A—C9A—N1A	122.9 (2)	C11—C10—C9	120.1 (3)
C11A—C10A—C9A	120.2 (3)	C11—C10—H10	119.9
C11A—C10A—H10A	119.9	C9—C10—H10	119.9
C9A—C10A—H10A	119.9	C12—C11—C10	120.5 (3)
C10A—C11A—C12A	121.1 (3)	C12—C11—H11	119.8
C10A—C11A—H11A	119.5	C10—C11—H11	119.8
C12A—C11A—H11A	119.5	C11—C12—C13	119.8 (3)
C11A—C12A—C13A	118.6 (3)	C11—C12—H12	120.1
C11A—C12A—H12A	120.7	C13—C12—H12	120.1
C13A—C12A—H12A	120.7	C14—C13—C12	120.3 (3)
C14A—C13A—C12A	120.9 (3)	C14—C13—C15	119.7 (4)
C14A—C13A—C15A	119.9 (3)	C12—C13—C15	119.9 (3)
C12A—C13A—C15A	119.2 (3)	C13—C14—C9	119.9 (3)
C13A—C14A—C9A	120.4 (3)	C13—C14—H14	120.0
C13A—C14A—H14A	119.8	C9—C14—H14	120.0
C9A—C14A—H14A	119.8	F1A—C15—F2B	130.2 (6)
F5B—C15A—F6A	76.3 (8)	F1A—C15—F3B	66.3 (8)
F5B—C15A—F4A	120.2 (8)	F2B—C15—F3B	94.1 (11)
F6A—C15A—F4A	103.8 (7)	F1A—C15—F2A	110.6 (6)
F5B—C15A—F4B	108.3 (10)	F3B—C15—F2A	124.9 (10)
F6A—C15A—F4B	124.8 (9)	F1A—C15—F3A	96.5 (8)
F5B—C15A—F6B	100.6 (9)	F2B—C15—F3A	68.9 (8)
F4A—C15A—F6B	86.0 (9)	F2A—C15—F3A	109.4 (8)
F4B—C15A—F6B	110.4 (11)	F1A—C15—F1B	47.0 (7)
F6A—C15A—F5A	105.9 (6)	F2B—C15—F1B	113.6 (8)
F4A—C15A—F5A	107.2 (6)	F3B—C15—F1B	111.0 (11)
F4B—C15A—F5A	87.0 (9)	F2A—C15—F1B	73.5 (8)
F6B—C15A—F5A	129.1 (8)	F3A—C15—F1B	136.9 (9)
F5B—C15A—C13A	118.0 (7)	F1A—C15—C13	116.7 (4)
F6A—C15A—C13A	115.0 (6)	F2B—C15—C13	113.1 (6)
F4A—C15A—C13A	115.4 (5)	F3B—C15—C13	115.3 (10)
F4B—C15A—C13A	110.4 (8)	F2A—C15—C13	113.9 (5)
F6B—C15A—C13A	108.6 (9)	F3A—C15—C13	108.2 (6)
F5A—C15A—C13A	108.9 (5)	F1B—C15—C13	109.3 (6)
C8A—N1A—C9A	121.1 (3)	C8—N1—C9	120.6 (3)
C2A—O1A—H1A	111 (3)	C2—O1—H1	106 (3)
C5A—O2A—C7A	118.1 (3)	C5—O2—C7	118.2 (3)
C6—C1—C2	118.6 (3)		
			
C6A—C1A—C2A—O1A	−180.0 (3)	C6—C1—C2—O1	−179.4 (3)
C8A—C1A—C2A—O1A	4.9 (4)	C8—C1—C2—O1	−2.7 (4)
C6A—C1A—C2A—C3A	0.8 (4)	C6—C1—C2—C3	−0.3 (4)
C8A—C1A—C2A—C3A	−174.3 (3)	C8—C1—C2—C3	176.4 (3)
O1A—C2A—C3A—C4A	−179.4 (3)	O1—C2—C3—C4	178.9 (3)
C1A—C2A—C3A—C4A	−0.1 (4)	C1—C2—C3—C4	−0.3 (4)
C2A—C3A—C4A—C5A	0.1 (5)	C2—C3—C4—C5	−0.2 (5)
C3A—C4A—C5A—C6A	−0.8 (4)	C3—C4—C5—O2	−177.9 (3)
C3A—C4A—C5A—O2A	178.2 (3)	C3—C4—C5—C6	1.2 (5)
O2A—C5A—C6A—C1A	−177.6 (3)	O2—C5—C6—C1	177.3 (3)
C4A—C5A—C6A—C1A	1.5 (5)	C4—C5—C6—C1	−1.8 (4)
C2A—C1A—C6A—C5A	−1.5 (4)	C2—C1—C6—C5	1.4 (4)
C8A—C1A—C6A—C5A	173.8 (3)	C8—C1—C6—C5	−175.4 (3)
C6A—C1A—C8A—N1A	−179.4 (3)	C6—C1—C8—N1	−179.0 (3)
C2A—C1A—C8A—N1A	−4.3 (4)	C2—C1—C8—N1	4.3 (4)
C14A—C9A—C10A—C11A	−1.1 (4)	C14—C9—C10—C11	0.8 (4)
N1A—C9A—C10A—C11A	−175.4 (3)	N1—C9—C10—C11	175.4 (3)
C9A—C10A—C11A—C12A	0.9 (5)	C9—C10—C11—C12	−0.7 (5)
C10A—C11A—C12A—C13A	0.1 (5)	C10—C11—C12—C13	0.3 (6)
C11A—C12A—C13A—C14A	−0.8 (5)	C11—C12—C13—C14	−0.1 (6)
C11A—C12A—C13A—C15A	177.8 (3)	C11—C12—C13—C15	−178.4 (4)
C12A—C13A—C14A—C9A	0.6 (5)	C12—C13—C14—C9	0.3 (5)
C15A—C13A—C14A—C9A	−178.0 (3)	C15—C13—C14—C9	178.5 (4)
C10A—C9A—C14A—C13A	0.4 (4)	C10—C9—C14—C13	−0.6 (5)
N1A—C9A—C14A—C13A	174.9 (3)	N1—C9—C14—C13	−175.5 (3)
C14A—C13A—C15A—F5B	166.6 (13)	C14—C13—C15—F1A	−18.4 (9)
C12A—C13A—C15A—F5B	−12.0 (14)	C12—C13—C15—F1A	159.9 (8)
C14A—C13A—C15A—F6A	−106.2 (9)	C14—C13—C15—F2B	163.3 (10)
C12A—C13A—C15A—F6A	75.2 (9)	C12—C13—C15—F2B	−18.5 (11)
C14A—C13A—C15A—F4A	14.6 (10)	C14—C13—C15—F3B	56.6 (12)
C12A—C13A—C15A—F4A	−164.0 (9)	C12—C13—C15—F3B	−125.1 (12)
C14A—C13A—C15A—F4B	41.4 (14)	C14—C13—C15—F2A	−149.1 (9)
C12A—C13A—C15A—F4B	−137.3 (13)	C12—C13—C15—F2A	29.1 (10)
C14A—C13A—C15A—F6B	−79.9 (13)	C14—C13—C15—F3A	89.0 (9)
C12A—C13A—C15A—F6B	101.5 (12)	C12—C13—C15—F3A	−92.7 (9)
C14A—C13A—C15A—F5A	135.2 (8)	C14—C13—C15—F1B	−69.1 (11)
C12A—C13A—C15A—F5A	−43.4 (9)	C12—C13—C15—F1B	109.1 (11)
C1A—C8A—N1A—C9A	170.5 (2)	C1—C8—N1—C9	−173.4 (2)
C14A—C9A—N1A—C8A	156.8 (3)	C14—C9—N1—C8	−149.7 (3)
C10A—C9A—N1A—C8A	−28.8 (4)	C10—C9—N1—C8	35.6 (4)
C6A—C5A—O2A—C7A	170.3 (3)	C6—C5—O2—C7	−165.4 (3)
C4A—C5A—O2A—C7A	−8.7 (5)	C4—C5—O2—C7	13.7 (5)

### Hydrogen-bond geometry (Å, °)

**Table:**

*D*—H···*A*	*D*—H	H···*A*	*D*···*A*	*D*—H···*A*
O1—H1···N1	0.91 (4)	1.79 (4)	2.619 (4)	150 (4)
O1A—H1A···N1A	0.87 (4)	1.87 (4)	2.623 (3)	143 (4)
C10—H10···O1^i^	0.93	2.58	3.444 (3)	154
C10A—H10A···O1A^i^	0.93	2.54	3.413 (3)	157
C3—H3···Cg3^ii^	0.93	2.86	3.526 (3)	130
C3A—H3A···Cg1^ii^	0.93	2.88	3.518 (3)	127
C11—H11···Cg4^iii^	0.93	2.85	3.529 (3)	131
C11A—H11A···Cg2^iii^	0.93	2.97	3.646 (3)	131

Symmetry codes: (i) *x*, *y*−1, *z*; (ii) −*x*+1, *y*+1/2, −*z*+1/2; (iii) −*x*+1, *y*−1/2, −*z*+1/2.
